# [Corrigendum] PVT1 promotes resistance to 5-FU in colon cancer via the miR-486-5p/CDK4 axis

**DOI:** 10.3892/ol.2026.15660

**Published:** 2026-05-20

**Authors:** Zhuhe Luo, Ruijun Chen, Shen Hu, Xibin Huang, Zhenyi Huang

Oncol Lett 24: 280, 2022; DOI: 10.3892/ol.2022.13400

Subsequently to the publication of the above paper, an interested reader drew to the authors' attention that, regarding the clone formation assay experiments shown in Fig. 6B on p. 11, the “Mimics NC” and “Mimics+ov+CDK4” images were apparently matching, suggesting that these data were incorporated into this figure erroneously.

Upon investigating their original data, the authors realized that the representative colony formation image selected for the “Mimics NC” group had mistakenly been reused for the “Mimics+ov-CDK4” group. The revised version of Fig. 6, now showing the correct data for the “Mimics+ov+CDK4” group in Fig. 6B, is shown on the next page. Note that this error did not seriously affect the conclusions reported in the paper. All the authors agree with the publication of this Corrigendum, and are grateful to the Editor of Oncology Letters for allowing them the opportunity to publish this Corrigendum; moreover, they apologize to the readership for any inconvenience caused.

## Figures and Tables

**Figure 6.**
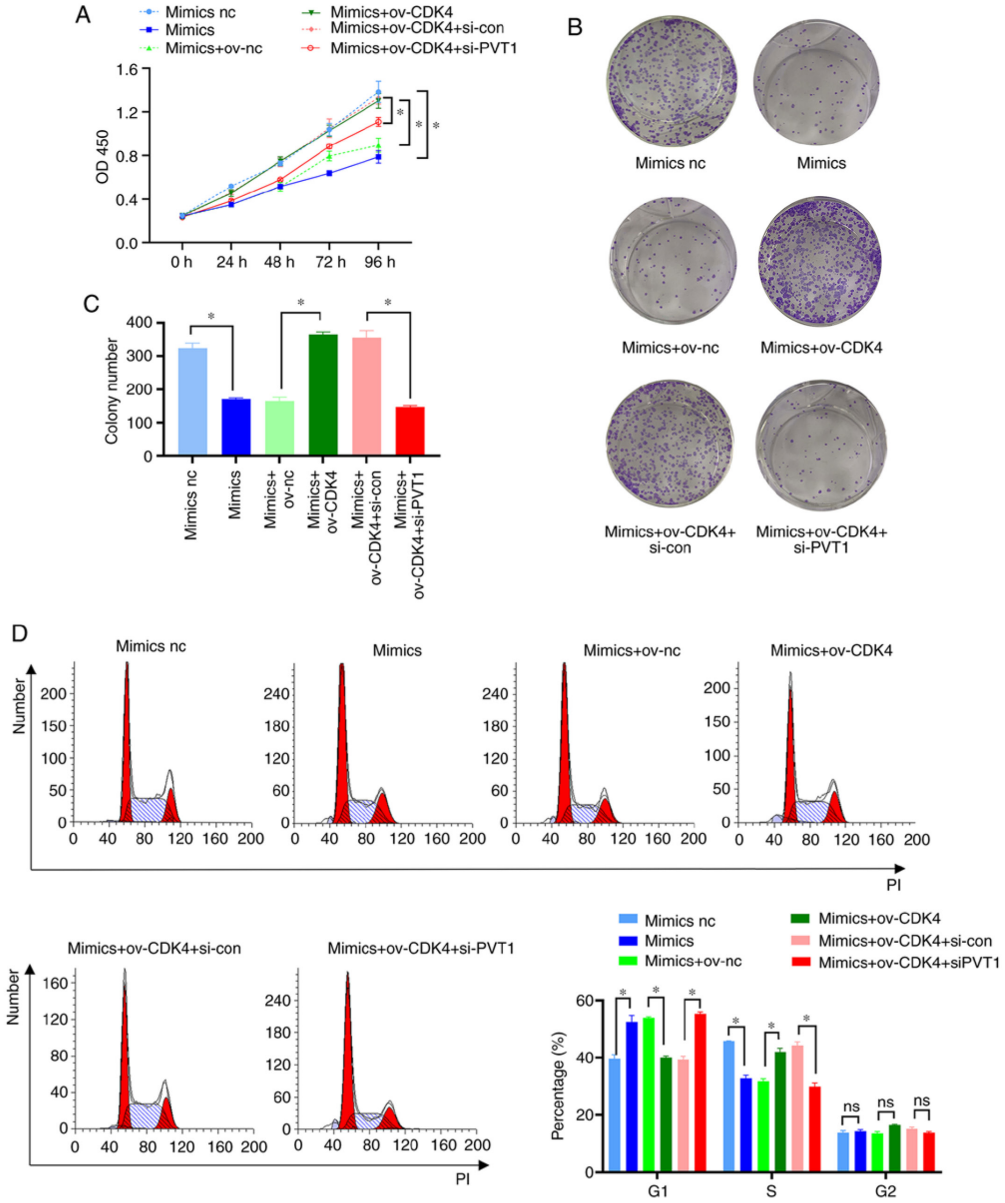
PVT1 regulates colon cancer cell 5-FU resistance via the miR-486-5p/CDK4 axis. (A) CCK-8 assay was used to detect the effects of the PVT1/miR-486-5p/CDK4 regulatory axis on the proliferation of HCT116-5FU-resistant cells. (B and C) A clone formation assay was used to assess the effects of the PVT1/miR-486-5p/CDK4 regulatory axis on clone formation by HCT116-5FU drug-resistant cells. (D) Flow cytometry was used to detect the effect of the PVT1/miR-486-5p/CDK4 regulatory axis on the cell cycle progression of HCT116-5FU drug-resistant cells. *P<0.05. PVT1, plasmacytoma variant translocation 1; 5-FU, 5-fluorouracil.

